# Post-transplant cyclophosphamide-induced cardiotoxicity: A comprehensive review

**DOI:** 10.34172/jcvtr.33230

**Published:** 2024-12-23

**Authors:** Azin Alizadehasl, Bita Shahrami, Reza Rahbarghazi, Azam Yalameh Aliabadi, Seyedeh Fatemeh Hosseini Jebelli, Yasamin Afsari Zonooz, Hoda Hakimian, Farzaneh Fathi, Sara Forati, Aysa Rezabakhsh

**Affiliations:** ^1^Cardio-Oncology Research Center, Rajaie Cardiovascular Medical and Research Center, Iran University of Medical Science, Tehran, Iran; ^2^Department of Clinical Pharmacy, School of Pharmacy, Tehran University of Medical Sciences, Tehran, Iran; ^3^Hematology, Oncology and Stem Cell Transplantation Research Center, Research Institute for Oncology, Hematology, and Cell Therapy, Tehran University of Medical Sciences, Tehran, Iran; ^4^Department of Applied Cell Sciences, Faculty of Advanced Medical Sciences, Tabriz University of Medical Sciences, Tabriz, Iran; ^5^Stem Cell Research Center, Tabriz University of Medical Sciences, Tabriz, Iran; ^6^Pharmaceutical Sciences Research Center, Ardabil University of Medical Sciences, Ardabil, Iran; ^7^Cardiovascular Research Center, Tabriz University of Medical Sciences, Tabriz, Iran

**Keywords:** Angiogenesis, Acrolein, Cardiotoxicity, Cyclophosphamide, Hematopoietic stem cell transplantation

## Abstract

Cyclophosphamide-induced cardiotoxicity, associated with its toxic metabolite acrolein, is a significant concern and unresolved issue, especially when cyclophosphamide is administrated in high doses. However, cardiotoxicity following low-dose cyclophosphamide has been also documented, especially in post-hematopoietic stem cell transplantation (post-HSCT) settings. Despite the involvement of multiple signaling pathways in cyclophosphamide-induced cardiomyopathy, the exact underlying mechanisms remain to be fully elucidated. This review outlines the current challenges of cyclophosphamide therapy in HSCT recipients. In addition, the promising therapeutic approaches by targeting acrolein’s anti-angiogenic effect were thoroughly discussed to better manage post-HSCT cyclophosphamide-induced cardiotoxicity.

## Introduction

 The irreversible cardiotoxicity induced by anti-neoplastic drugs such as anthracyclines and alkylating agents is considered a life-threatening health issue in the clinical setting.^1,2^ According to the International Agency for Research in Cancer (IARC) declaration, cyclophosphamide, an alkylating cytotoxic agent,^3^ possesses a chemotropic effect used in various types of cancers, including neuroblastoma, endometrial, breast, and lung cancers, as well as malignancies such as leukemia, lymphoma, multiple myeloma, and immune-mediated conditions, like rheumatoid arthritis (RA), multiple sclerosis (MS), and life-threatening anti-neutrophil cytoplasmic antibodies (ANCA)-associated vasculitides, and even connective tissue diseases such as systemic lupus erythematosus (SLE) or scleroderma (SSc).^1,4,5^ Due to immunosuppressive properties, cyclophosphamide is also utilized in hematopoietic stem cell transplantation (HSCT) to regulate immune-reactive cells.^6,7^ Despite these advantages, the application of cyclophosphamide is accompanied by multiple adverse events reported in several organs primarily cardiac tissue. Arrhythmias, myocardial infarction (MI), atrioventricular block, thromboembolism, congestive heart failure (CHF), hemorrhagic cell necrosis, and fatal myopericarditis have been reported in patients receiving cyclophosphamide.^8,9^

 It is believed that cyclophosphamide-induced cardiotoxicity is a dose-dependent phenomenon, and the clinical manifestations are common following the application of high doses, ranging between 75 - 200 mg/kg.^10,11^ Declined amplitude of the QRS complex, non-specific T-wave abnormalities, and asymptomatic reduction of left ventricular ejection fraction (LVEF) are diagnosed in patients within two weeks after cyclophosphamide administration.^2,12^

 Noticeably, anti-angiogenic agents like acrolein in cyclophosphamide structure, are also associated with several cardiovascular disorders, including hypertension, LV systolic dysfunction (LVSD), HF, arterial/venous thromboembolism, arrhythmia, and QT prolongation.^13^

 As therapeutic approaches, adjusting cyclophosphamide dose and administration period, monitoring elimination/excretion, and boosting the endogenous antioxidant status could be considered to reduce subsequent complications.

 The main aim of this review is to present the current evidence regarding the challenges in the face of post-HSCT cardiac sequelae induced by cyclophosphamide administration to further discuss presumable solutions maybe through targeting acrolein’s anti-angiogenic properties in the clinical setting.

## Methods

###  Search strategy

 The scientific literature regarding cyclophosphamide-induced cardiotoxicity was extensively performed using various databases, including PubMed Central, Embase, Cochrane Central Register of Controlled Trials, and preprint servers (i.e., medRxiv) up to June 2024. In addition, keywords resulting from the Medical Subject Headings (MeSH) tool were considered as follows:

 “ Cyclophosphamide” OR “ 3-(1-oxy-2,2,6,6-tetramethyl-4-piperidinyl) cyclophosphamide “ OR “ d4-cyclophosphamide “ OR “ 4-hydroxyperoxycyclophosphamide “ OR “ N(3)-methyl-4-(propylthio)cyclophosphamide “ OR “Alkylating agents” OR “ALKYLATING AGENTS “

 AND

 “ Cardiotoxicity “ OR “ Cardiomyopathy “

 AND

 “ Hematopoietic Stem Cell Transplantation “ OR “ Bone Marrow Transplantation “

###  Inclusion/exclusion criteria

 All published articles that were scientifically relevant to the scope of our research, consisting of randomized clinical trials (RCTs), observational studies, systematic reviews and meta-analyses, case reports, and letters to the editor were included. While, unpublished and non-English written articles, as exclusion criteria, were not eligible to be included in this study.

## Cyclophosphamide Attributes

###  Pharmacokinetics and pharmacodynamics properties 

**Figure 1:**
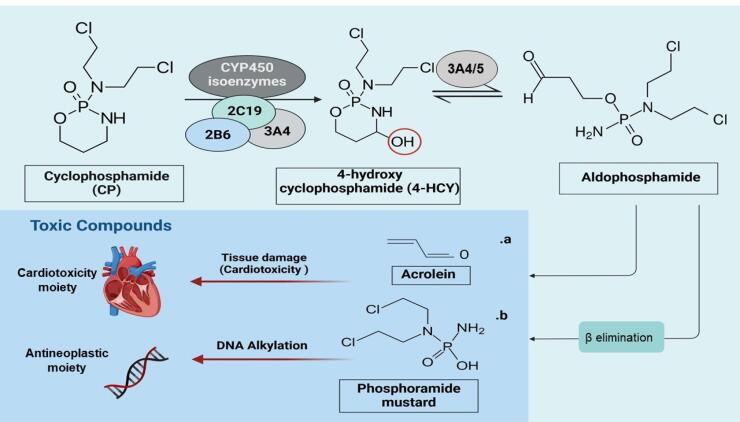
Pharmacokinetics properties of cyclophosphamide administration. **Abbreviations:** 4-HCY, 4-hydroxy cyclophosphamide; CYP450, Cytochrome P450

 Cyclophosphamide, as a prodrug belonging to the oxazaphosporines group, is metabolized in the hepatic tissue and converts to two byproducts including 4-hydroxy Cyclophosphamide (4-HCP) and aldophosphamide via the activity of various cytochrome p450 (CYP450) isoenzymes, such as CYPs 3A4, 2B6, 2C19.^14,15^ The further structural modification produces phosphoramide mustard, an ultimate alkylating agent, with an anti-neoplastic activity.^14,15^ Following oral or parenteral administration, cyclophosphamide undergoes hepatic biotransformation and further converts into non-toxic metabolites, such as carboxyphosphamide, nitrogen mustard, and reactive aldehyde, named acrolein, which is responsible for promoting cytotoxic effects on myocardium and endothelial cells (ECs), as well as hemorrhagic cystitis^16^ (Figure 1).

 In a study designed to evaluate the pharmacokinetics properties of 5-75 mg/kg of cyclophosphamide in patients aged less than two years old, a marked difference in clearance rate, likely due to pharmacogenomics differences, was found regardless of a substantial disparity in dosing protocols when compared to the older population.^17^

 Due to the generation of acrolein following cyclophosphamide administration, the rate of CVD risk can be monitored by measuring the urine level of 3-hydroxypropyl mercapturic acid (3-HPMA) which is the acrolein’s major metabolite.^18^ In an *in vitro* assessment, cytotoxic effects caused by various cyclophosphamide metabolites were evaluated in rat cardiomyoblast H9C2 cells. Data showed that 4-HCP and acrolein, but not carboxyethylphosphoramide mustard (CEPM), are the main key mediators in the production of reactive oxygen species (ROS) and cardiomyocyte toxicity following cyclophosphamide treatment.^19^ In the animal model, the protective impact of aldehyde dehydrogenase (ALDH), an endogenous cardioprotective enzyme, in restoring the function of inured cardiomyocytes is significantly mitigated.^19^

 Furthermore, some drugs exacerbate cyclophosphamide-related cardiotoxicity through modulation of drug metabolism. For example, antifungal medications with the azole group, as potent inhibitors of CYP2B6, interfere with cyclophosphamide hepatic metabolism and subsequently lead to higher serum levels of cyclophosphamide, and, in turn, enhance the possibility of related toxicities.^8^

 At the cellular level, cyclophosphamide can covalently bind to the proteins or DNAs via alkyl moieties, resulting in the generation of ROS and subsequent apoptotic cell death.^20^ These features increase the possibility of some hematologic disorders, including anemia, neutropenia, thrombocytopenia, BM suppression, bladder toxicity, and gonadal failure.^13^ As above-mentioned, a high dose of cyclophosphamide (100 mg/kg <) is also administered in patients subjected to BM transplantation, while lower doses are recommended in hematologic and non-hematologic malignancies.^13^ However, available evidence corroborated that the onset of cardiotoxicity symptoms appears as early as 48 hours after administration of cyclophosphamide > 150 mg/kg or values more than 1.55 g/m^2^ per day.

## Cyclophosphamide-induced cardiotoxicity in Chemotherapy, Hematopoietic Stem Cell Transplant, and Non-Cancer treatment setting

 In the field of cancer therapy, cyclophosphamide-induced cardiotoxicity is a significant concern, particularly in high-dose regimens. Excessive oxidative/nitrosative status and direct damage to the ECs lead to conditions like cardiomyopathy and heart failure. Post-transplant cyclophosphamide administration, as a part of the conditioning regimen, is common in patients with haploidentical HSCT to prevent possible graft-versus-host disease (GVHD) and subsequently increase survival rates. However, this high-dose usage enhances the risk of cardiotoxicity, manifesting most likely as hemorrhagic myocarditis or pericarditis.^21^ Moreover, cardiotoxicity induced by cyclophosphamide in non-cancer settings, such as autoimmune diseases, remains a risk, particularly following prolonged or high-dose treatments.

###  Epidemiological reports

 Despite existing limited data to determine cardiotoxicity prevalence, regarding the long-term cardiotoxicity occurrence upon the course of chemotherapy, a previous report estimated a higher rate of HF (15‐fold), CVDs (10‐fold), and stroke (9‐fold) occurrence, respectively.^22^ Noteworthy, according to the epidemiological analysis, the risk of heart failure has been also estimated between 7-28% following cyclophosphamide treatment.^23^ Meanwhile, post-transplant cyclophosphamide application can predispose patients to the risk of cardiac events and increase ~1.5 to 3.5-fold mortality rates.^24^ Also, secondary to post-HSCT cyclophosphamide administration, the incidence of acute heart failure and mortality rate has been estimated at 20% and 8%, respectively.^22^ Regardless of the multiple factors involved in cardiotoxicity development, another report indicated that in patients who receive a combination therapy of anthracyclines and cyclophosphamide, the estimated incidence of cardiotoxicity rises to 28%.^25^

###  Etiology 

 Regarding the defined etiologies to develop cardiotoxicity, exposure to chemicals such as metals, environmental pollutants, oxidative agents, radiation therapy, and medications, i.e., some chemotherapy and immune-suppressive drugs have been documented, resulting in a higher rate of morbidity and mortality worldwide.^26^

## Pathophysiology of cyclophosphamide-induced cardiotoxicity

 Although the exact mechanisms related to cyclophosphamide-induced cardiotoxicity have not been fully addressed, it has been assumed that the direct injury of ECs and myocytes can be initiated in response to excessive production of toxic metabolites such as ROS and further oxidative/nitrosative endoplasmic reticulum (ER) stress upon cyclophosphamide administration, generating prominent histopathological changes. In detail, the direct injury of microvascular ECs increases the chance of close exposure of toxic metabolites with cardiomyocytes in deep layers of the myocardium, contributing to massive interstitial edema, intravascular thrombus, thickness of LV wall and interventricular septum, and hemorrhagic myocarditis-pericarditis.^21,24,27^ Due to the development of coronary vasospasm and microvascular emboli following cyclophosphamide treatment, the occurrence of ischemic myocardium is mighty.^27^

###  Molecular mechanisms of cyclophosphamide-induced cardiotoxicity 

 According to a gear body of scientific data, various singling pathways are involved in the pathogenicity of cyclophosphamide metabolites-derived cardiotoxicity. Cyclophosphamide has the potential to promote various signaling pathways involved in oxidative/nitrosative stress and inflammatory responses, leading to redox imbalance and intracellular damages, such as sarcoplasmic reticulum dilatation, mitochondrial abnormality, and nuclear membrane deformation.^28,29^ Here, we abridged the molecular mechanisms involved in cardiomyopathy progression following cyclophosphamide administration.

###  Oxidative stress and inflammatory responses

 Cyclophosphamide can provoke an inflammatory reaction in cardiac tissue and predispose the cardiomyocytes to further degenerative changes.^30^ The inflammatory response following cyclophosphamide administration can be induced during three main stages: 1) Cyclophosphamide and related metabolites are able to increase nitrosative/oxidative stress by robust ROS generation and nitrogen reactive species (RNS) through the activation of inducible and endothelial nitrogen synthase (iNOS and eNOS), 2) by activation of Toll-like receptor 4 (TLR-4) and engaging intracellular p38-mitogen-activated protein kinase (p38MAPK) and c-Jun N-terminal kinases (JNK) axis inside the sensitize cardiomyocytes to cyclophosphamide-associated injuries. At the same time, cyclophosphamide initially enhances the levels of peroxynitrite, and inflammatory responses through p38MAPK and JNK signaling pathway activation. Furthermore, cyclophosphamide can inhibit the mitogen-activated protein kinase (MEK1/2) axis with the potential to cause cardiomyocyte toxicity via mitochondrial injury. Following the activation of TLR-4, the expression of various pro-inflammatory mediators such as TNF-α, interleukins (ILs), especially IL-1β, and cyclooxygenase-2 (COX-2) is increased. In turn, 3) nuclear factor (NF)-κB is translocated into the nuclei and stimulates the production of pro-inflammatory cytokines, as well. Along with these changes, cellular contents of ATP are reduced because of mitochondrial dysfunction, leading to energy imbalance further intracellular calcium (Ca^2+^) accumulation, and cardiomyocyte injury.^28^

 In a study conducted by Hassanein et al, the therapeutic impact of lansoprazole against cardiopulmonary injury induced by oxidative stress and inflammatory response following cyclophosphamide administration was evaluated ^31^. Surprisingly, the results of this study unveiled a protective effect of lansoprazole against elevated redox status in cardiac tissue through modulating PPARγ, Nuclear factor erythroid 2-related factor 2 (Nrf2)/ Heme Oxygenase-1 (HO-1), cytoglobin, PI3K/AKT, and NF-κB signaling pathways.^31^

###  Cardiomyocyte apoptosis 

 Accumulating data have shown an apoptotic potential of cyclophosphamide in preclinical settings.^32-34^ To be specific, the levels of pro-apoptotic factors mainly belonging to the Bcl-2 family protein, *i.e.*, Bcl-2-associated X-protein (BAX) and Bcl-2-associated death (BAD) promoters are increased. Along with these changes, the release of cytochrome C (Cyt c) from mitochondria is stimulated. Released Cyt c engages apoptotic-activated protease factor-1 (Apaf-1) to expedite the apoptosome formation, and activate Caspases 3 and 9. Some data have also indicated that cyclophosphamide can increase protein kinase B (PKB) and glycogen synthase kinase-3 beta (GSK-3β) levels. Of note, the increased PKC activity and inositol triphosphate (IP3) levels heighten intracellular ionized calcium (Ca^2+^).^35^

###  Cardiomyocytes’ energy pool modulation 

 Following cyclophosphamide treatment, the expression of heart fatty acid binding protein (H-FABP) is downregulated, inhibiting free fatty acid (FFA) transportation into the mitochondria. Furthermore, cyclophosphamide affects the β-oxidation levels in the mitochondrial matrix, which is mediated by malonyl-CoA increasing, derived from acetyl CoA, and carnitine palmitoyl transferase-I (CPT-1) decreasing, ultimately resulting in cytosol FFA and Ca2 + accumulation.^28^ Therefore, the energy balance and ATP production are also disturbed by cyclophosphamide exposure.

###  EC dysfunction and cardiomyopathy 

 Noteworthy, cyclophosphamide administration converts normal fibroblasts into the abnormal phenotype named myofibroblasts (myoFBs), which accelerate the cardiac injury through NADPH oxidase activation and subsequently increase the pro-inflammatory cytokines, leukotrienes, and oxidative stress to finally develop cardiac fibrosis. Moreover, EC dysfunction induced by cyclophosphamide is mediated by the reduction of nitric oxide (NO) synthesis and endothelial progenitor cell production.^36^ On the other hand, increased expression of endothelin-1, NF-κB, Platelet factor 4 (PF4), and monocyte adhesion, in turn, participate in vasoconstriction and subsequently lead to hypertension incident.^37^

###  Left ventricle remodeling and cardiac hypertrophy 

 Following the redox imbalance and extra intracellular accumulation of Ca2 + , the apoptosis activation occurs through the caspase/BAX axis. Simultaneously, p38MAPK/ c- JNK downstream and p53 activation can be observed following TLR-4 stimulation, accompanied by the pro-inflammatory cytokines upregulation (TNF-α, and IL-1β), which conclusively promotes nucleic anti-inflammatory Nrf2/ARE suppression. Ultimately, apoptosis and inflammatory response under oxidative/nitrosative conditions cause cardiac hypertrophy and LV remodeling.^38^

## Clinical Outcomes of post-transplant cardiotoxicity induced by cyclophosphamide

 Despite early cardiovascular adverse events (within the first 100 days) associated with post-transplant cyclophosphamide administration being rare, available evidence reported a strong association between cyclophosphamide administration and clinical outcomes with a hazard ratio of 2.7; and a 95% confidence interval of 1.4 to 4.9 (*P* = 0.002). The complications encompass LV systolic dysfunction, acute pulmonary edema, pericarditis, arrhythmia, and acute coronary syndrome (ACS) followed by a lower overall survival rate.^24^ In parallel with this study, Ishida et al also elucidated the diastolic dysfunction (based on E/A ratio) induced by > 100 mg/kg of cyclophosphamide after a conditioning regimen of allogeneic HSCT (allo-HSCT),^39^ which can subsequently cause a dose-dependent fatal cardiac failure.^40^ Noteworthy, despite acceptable screening of cardiopulmonary issues, high-dose cyclophosphamide administration could promote fatal cardiotoxicity sequela

 To ascertain low-dose cyclophosphamide-induced arrhythmogenicity at the cellular levels (below 100 mg/kg), Podgurskaya et al assessed the functional (conduction properties) and structural changes (cytoskeleton organization) of human iPSC-derived cardiomyocytes (hiPSC-CMs) after treatment with cyclophosphamide.^3^ They found that conduction parameters, including maximum capture rate (MCR) and conductivity area in hiPSC-CMs, were significantly diminished via the disruption of alpha-actinin following cyclophosphamide-induced arrhythmogenicity (25% ± 7% and 34 ± 15%, respectively).^3^ Finally, they revealed that deviation from the external stimulus frequency and appearance of non-conductive areas can be considered two major factors involved in cyclophosphamide-induced arrhythmogenicity *in vivo*.^3^ Moreover, a previously report showed some rare cyclophosphamide-induced cardiomyopathy lethal complications (≥ 200 mg/kg) among 811 allo-HSCT recipients, including fatal HF (1.5%), and a high mortality rate (91.6%).^41^ However, a significant fatal HF was not found in individuals who received 100 mg/kg post-transplant cyclophosphamide regimen, underscoring that early diagnosis and close-up monitoring in the ICU setting would be great aids in optimizing clinical outcomes.^41^

 Notably, a recent study also explored the clinical properties of patients admitted in ICU settings due to the cardiotoxicity following cyclophosphamide administration. The most observed complications observed among cyclophosphamide recipients were acute pulmonary edema, cardiac arrest, and cardiac arrhythmia, respectively.^42^ The most common echocardiography findings were also related to systolic dysfunction and pericardial effusion in this subset of patients.^42^ The findings of this study elegantly revealed that refractory cardiogenic shock was strongly associated with the mortality rate in patients with severe forms of cardiotoxicity.^42^ In Table 1, post-transplant cyclophosphamide-induced cardiotoxicity in various stem cell recipients has been brought.

**Table 1:** Studies in the setting of -cyclophosphamide-induced acute cardiotoxicity in cell transplant recipients

**Study**	**Disease**	**Patients**	**Cell type**	**Dose of CY**	**Cardiotoxicity**	**Assessment tool and related indices**	**Outcomes**
Podgurskaya et al, 2021^3^	Arrhythmo-genicity	*In vitro *	hiPSC-CMs	213–852 µM	MCR and conductivity area decreased. alpha-actinin disruption (cytoskeleton structure)	Optical mapping method to measure CV, MCR, and number of occasions of re-entry on a standard linear obstacle	Cyclophosphamide appeared to be a potential arrhythmogenic agent in the experimental setting.
Duléry et al, 2021^24^	AML, ALL, Lymphoma, MM, MDS, MPN	331 patients, PT- cyclophosphamide (n = 136, 86 = males, 50 females) and non-PT-Cy (n = 195, 114 males, 81 females), 15 ≤ years old	Allo-HSCT	Low dose:50 mg/kg/day	ECE**	Transthoracic Echo	The incidence of ECE** was significantly higher in PT-cyclophosphamide patients (*P* < 0.001) with lower overall survival (HR: 2.7; 95% CI: 1.4 to 4.9; *P* = 0.002)
Ishida et al, 2015^39^	LM, including SAA, MM, Lymphoma, AML, CML, MDS	811 patients	Allo-HSCT	High dose: 120 (60 mg for two days), A combination therapy with lymphoid irradiation and/or busulfan	Fatal HF, Diastolic dysfunction	2D echo, E-wave, A-wave, E/A index, LVEF	Due to fatal cyclophosphamide-induced cardiotoxicity which is associated with a high mortality rate, it must be considered early after the administration of the drug
Zver et al, 2008^43^	MM	30 patients, (19 males and 11 females), 40-70 years old	ASCT	High dose of Cy (133.33 mg/kg) + MEL (6.66 mg/ kg) Myeloablative dose* 2x) for 2 weeks	Mitral regurgitation, Diastolic LV dysfunction	Echo, Conventional, and Doppler to measure E_m_, a-wave, and A/a ratio and inflow velocities	Following tandem autologous HSCT cardiotoxic effects with no clinical signs of HF was detected.
Ma et al, 2023^44^	SAA	17 patients (7 = males, 10 = females), < 40 years old	Haplo-HSCT	High dose: Total 200 mg/kg (divided into 42.75 mg/kg four days before HSCT, and 14.5 mg/kg for two days after HSCT)	Grade II	-	Acceptable survival outcomes and reduced incidence of GVHD were achieved

* High-dose chemotherapy to destroy cancer cells in the bone marrow. ** ECE is defined as LVSD, decreased LVEF, acute pulmonary edema, arrhythmia, pericarditis, or ACS during the first 100 days. **Abbreviations:** 2-dimensional echo, 2D Echo; ACS, acute coronary syndrome; Allo-SCT, allogeneic stem cell transplantation; ASCT, autologous stem cell transplantation; Acute lymphoblastic leukemia; AML, Acute myeloid leukemia; Auto-HCT, autologous hematopoietic stem cell transplantation; BNP, Brain natriuretic peptide; CI, confidence interval; CV, conduction velocity; ECE, Early Cardiac Events; Eco, Echocardiography; ET-1, Endothelin 1; LVEF, Left Ventricular Ejection fraction; LVSD, left ventricular systolic dysfunction; haplo-HSCT, Haploidentical hematopoietic stem cell transplantation; HF, heart failure; HiPSC-CMs, human induced pluripotent stem cell-derived Cardiomyocytes; HSCT, haploidentical hematopoietic stem cell transplantation; HR, hazard ratio; LM, Lymphoid malignancy; MCR, maximum capture rate; MDS, Myelodysplastic syndrome; MEL, Melphalan; MM, Multiple Myeloma; MPN, Myeloproliferative neoplasm; PT-Cy, Post-transplant cyclophosphamide; SAA, Severe Aplastic Anemia; TnI, Troponin I

**Figure 2:**
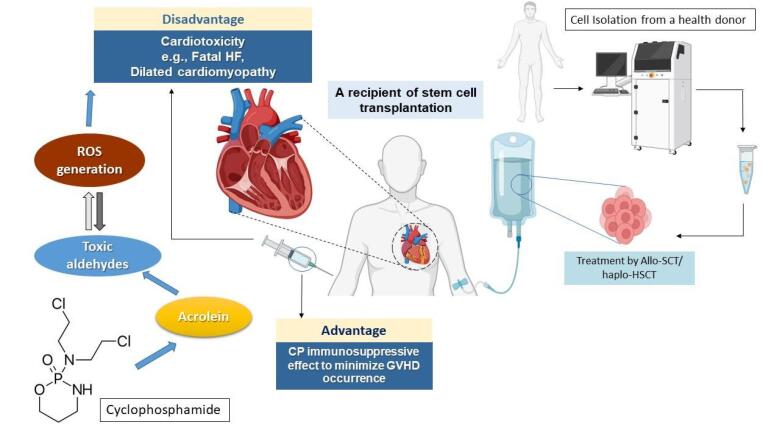
A schematic presentation ofcyclophosphamide-induced cardiotoxicity following HSCT.
**Abbreviations:** Allo-SCT, allogeneic stem cell transplantation; GVHD, graft-versus human disease; Haplo-HSCT, haploidentical hematopoietic stem cell transplantation; HF, heart failure; ROS, reactive oxygen species

 Regarding the assessment of laboratory parameters after cyclophosphamide exposure, serum levels of BNP and endothelin-1 (ET-1) markedly increased in patients with multiple myeloma undergoing tandem autologous HSCT.^45^ Although significant clinical manifestations in favor of HF and myocardial necrosis were not observed, cardiac LV dysfunction and mitral regurgitation were approved by echocardiography indices, including wave prolongation in the pulmonary vein flow, A/a ratio reduction in flow velocities, and decreased early diastolic tissue Doppler velocities.^45,46^ In Figure 2, the clinical outcomes of cyclophosphamide-induced cardiotoxicity in HSCT recipients were schematically illustrated.

## Predisposing Factors

 Several risk factors such as pre-existing ischemic heart disease, a history of chest radiation therapy (especially mediastinum or left chest wall), and simultaneous treatment with other cardiotoxic agents, e.g., anthracyclines, should be considered main risk factors prior to cyclophosphamide administration in HSCT recipients.^47^ Besides, it has been well-established that high-dose cyclophosphamide, and some predisposing conditions such as older ages ( > 55 years old), metabolic disorders, e.g., diabetes mellitus, and receiving heavy pre-treatment regimens can increase the likelihood of cardiotoxicity.^8^ According to a recent study conducted by LeMaistre et al, it has been also found that the occurrence of severe LVEF reduction in patients who received post-transplant cyclophosphamide is more frequent in those with a history of systolic dysfunction without worsening survival outcomes.^48^

## Therapeutic Strategies to Manage Cyclophosphamide-Induced Cardiotoxicity

 Non-fatal cardiomyopathy is reversible and the symptoms can be managed by prescribing some antihypertensive medications such as beta-blockers, diuretics, angiotensin-converting enzyme inhibitors (ACEIs), angiotensin II receptor blockers (ARBs), vasodilators, and inotrope drugs followed by appropriate hemodynamic support and close monitoring.^8^ Recently, a cardioprotective effect of simvastatin against cyclophosphamide-induced toxicity has been also evaluated. The *in vivo* study results showed that simvastatin can exert appreciated anti-inflammatory and anti-apoptotic effects via NLRP3-inflammasome/ caspase 1/ IL1β pathway modulation.^49^ In this regard, eNOS also plays a crucial role in alleviating cyclophosphamide-induced cardiomyopathy mediated by simvastatin.^49^

 An experimental study also indicated the ALDH2 crucial role in reducing the sensitivity against cyclophosphamide-induced acute cardiotoxicity, and cardiomyocyte necrosis, as well as accumulation of ROS, acrolein, and other toxic aldehyde metabolites.^50^ Besides, the therapeutic potential of numerous natural products with potent anti-oxidant effects was also widely investigated in preclinical settings.^38^

## Prevention of cyclophosphamide-induced Cardiotoxicity

 Considering an alternative therapeutic protocol would be the utmost strategy to prevent the occurrence of fatal cardiotoxicity in the presence of the above-mentioned risk factors. However, early diagnosis of cyclophosphamide-induced cardiotoxicity plays a crucial role in preventing worsening outcomes.

 BuCyFlu conditioning regime consisting of busulfan (Bu, 3.2 mg/kg), low-dose cyclophosphamide (60-100 mg/kg), fludarabine (150 mg/m2), and rabbit anti-thymocyte globulin (ATG) (10 mg/kg) has been utilized after haplo-HSCT to control cardiac complications in patients with severe aplastic anemia.^19^ This regimen can contribute to restricting cyclophosphamide-induced severe cardiomyopathy (2.17% *vs.* 12.80%, *P* < 0.05) with favorable survival and engraftment outcomes when compared to high-dose cyclophosphamide counterparts.^51^ In this line, a recent prospective clinical trial also reported that the order of cyclophosphamide and busulphan-based treatment before receiving HSCT substantially minimized organ toxicity (CyBu superior to BuCy).^52^

 In patients with severe aplastic anemia (SAA) undergoing haplo-HSCT, modified granulocyte colony-stimulating factor/anti-thymocyte globulin-based protocol of cyclophosphamide treatment can be also used to reduce the unfavorable cardiac events along with the increased engraftment efficiency.^44^ A large number of SAA patients with novel regimens, *i.e.*, splitting of cyclophosphamide high dose (200 mg/kg) into separate doses (42.75 and 14.5 mg/ kg), exhibited acute GVHD grades I-II but not III-IV grades with non-fatal cardiotoxicity (grade II) and prolong survival rate during follow-up.^44^

###  Cardiotoxicity management by targeting acrolein-induced anti-angiogenic effect

 Angiogenesis is defined as a pathophysiological process to form new capillaries out of pre-existing vasculature.^53,54^ In this regard, out of various identified factors, vascular endothelial growth factors (VEGFs) play a major role in driving neovascularization.^55^ In addition, numerous modulators such as nitric oxide (NO), HO-1, ROS, and other inflammatory molecules also participate in deriving vasculogenesis under pathological conditions.^56^ In the case of cross-link between impaired angiogenesis and cardiotoxicity development, a recent study emphasized a direct association between selected VEGF-receptor tyrosine kinase inhibitors (VEGF-TKI) and cardiotoxicity induction in patients with malignancy.^57^

 Pro-angiogenic target therapy is being recently employed as a cutting-edge therapeutic approach in the field of ischemic CVD. In this sense, some anti-angiogenic agents with specific targets, causing aberrant neovascularization and increasing vascular permeability have been introduced.^58^ For instance, a spermine oxidase inhibitor, named MDL 72527, serves a potential anti-angiogenic effect through acrolein-conjugated protein reduction and P38/ERK1/2/STAT3 signaling pathway downregulation.^58^

 However, some angiogenic inhibitors, such as monoclonal antibodies (mAbs) and TKIs, are also associated with cardiovascular adverse events, including HF, MI, stroke, and venous thromboembolism,^59,13^ which further highlight the substantial role of acrolein, as a toxic byproduct, in promoting organ damage.

 In addition to the DNA alkylation mediated by phosphoramide mustard in tumor cells, cyclophosphamide also exerts a double tumor therapy effect through unsaturated aldehyde, acrolein, most likely during thrombospondin-1 (TSP-1) upregulation, an endogenous angiogenesis inhibitor, to further restrict tumor cells proliferation and migration.^60^ A previous study conducted on smokers has confirmed that acrolein is directly associated with increased CVD risk via the promotion of platelet aggregation and reduction of circulating early and late angiogenic cells (ACs).^18^

**Figure 3:**
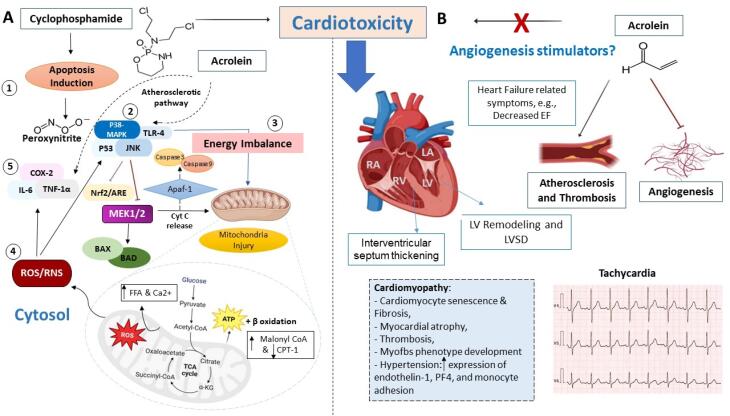
Cyclophophamide-induced cardiotoxicity. **A.** involved mechanism of action following: (1) apoptosis, (2) activation of TLR-4-MAPK-JNK signaling pathway and subsequent (3) energy imbalance and mitochondrial damage, (4) increased ROS generation and (5) activation of inflammatory response mainly mediated by TNF-α, IL-6, and COX-2. **B.** Cardiotoxicity derived by Acrolein anti-angiogenic effect.
**Abbreviations:** APAF-1, Apoptotic Protease Activating Factor 1; ATP, Adenosine triphosphate; BAD, BCL2 associated agonist of cell death; BAX, Bcl-2-associated X-protein; Caspase, cysteine-dependent, aspartate-specific peptidase; CoA, coenzyme A; COX-2, cyclooxygenase-2; CPT-1, Carnitine palmitoyltransferase 1; EF, ejection fraction; FFA, free fatty acid; HF, heart failure; JNK, c-Jun N-terminal kinases; IL-6, interleukine-6; LA, left atrial; LV, left ventricular, LVSD, left ventricular systolic dysfunction; MAPK, mitogen-activated protein kinase; RA, right atrial; RV, right ventricular; ROS, reactive oxygen species; RNS, reactive nitrogen species; TLR-4, toll-like receptor-4; TNF-α, Tumor necrosis factor-alpha

 Beyond the anti-angiogenic property, it has been well-established that acrolein can also appear to be an atherosclerotic factor due to the increase of the inflammatory responses via cyclooxygenase-2 (COX-2) upregulation and prostaglandins (PGs) production through PKC- p38MAPK signaling pathway *in vitro*.^57^ Hence, some proposed anti-inflammatory therapy with natural products, such as curcumin, would be a promising adjuvant therapy to improve vascular performance and subsequent alleviation of cyclophosphamide-induced cardiotoxicity.^61^ Another option against acrolein-induced cytotoxicity is referred to the acrolein trapping with active flavonols, possessing abundant phenolic –OH groups in structural B-ring (not A- and C-ring), especially myricetin, to scavenge the toxic acrolein.^62^ In better words, myricetin by acrolein trapping results in non-toxic acrolein adduct formation (mono- and di-ACR-myricetin adducts) in a dose-dependent manner.^62^ In Figure 3, the negative effects of acrolein on cardiac tissue following cyclophosphamide administration were described.

## Discussion

 In this review, we primarily discussed the pharmacologic features of cyclophosphamide and the pathogenesis of possible cardiotoxicity. Next, potential therapeutic approaches such as targeting of anti-angiogenic effect of acrolein in HSCT recipients were proposed for upcoming research.

 To the best of our knowledge, cardiotoxicity refers to the early and late incidence of either cardiac electrophysiology dysfunction or myocardia weakness. According to the Cardiac Review and Evaluation Committee declaration, the cardiotoxicity definition mainly refers to either LVEF reduction between 5-55% with CHF symptoms or LVEF reduction between 10-55% without clinical symptoms,^9,29^ which can influence the optimal impact of current anti-cancer or immunosuppressive therapies used short- or long-term. It is worth noting that beyond the undesirable cardiotoxicity, patients undergoing cyclophosphamide therapy are also at higher risk of other adverse effects like gonadal toxicity, bone marrow suppression, and even secondary malignancy due to cumulative dose effects.^63^

 Under allogeneic stem cell transplantation, some therapeutic strategies should be applied to reduce the activation of all-reactive immune cells such as immunocompromised drugs. In this respect, post-transplant cyclophosphamide therapy has been defined as a standard of care, particularly in individuals receiving haploidentical HSCT (haplo-HSCT) with the purpose of GVHD reduction risk.^64^ It is also common in human leukocyte antigen (HLA)-identical sibling, matched-unrelated, and mismatched unrelated donor HSCT.^65-67^ In patients undergoing HSCT, cyclophosphamide is generally administered at a dose of 100 mg/kg and above, while in patients with hematological malignancies or solid tumors, these values are in the range of 80-200 mg/kg.^9^ However, the precise dose and administration period can be varied according to the treatment type, and concomitant therapeutic protocols.^10^

 Following cyclophosphamide administration, it has been well-documented that cardiomyocyte death, which emerged as the leading cause of long-term irreversible cardiac damage, is mainly mediated by vasculature dysfunction and thromboembolic ischemic events. However,alkylating agents may also cause type II or reversible cardiotoxicity without inducing cell death,which is associated with a lower rate of cardiomyocyte impairment and HF incidents. Mechanistically,it has been also assumed thattype II cardiotoxicitymay occursecondary toeitherthederegulation of cardiomyocyte-intrinsic mechanismsor extracellular factors,especially paracrine factors to modify cardiomyocyte function.Due to the limited potential of cardiomyocyte regeneration in the adult population on the one hand,andthe broad array of biological mechanismsaffected bycyclophosphamide and related toxic metabolites, on the other hand,the development of novel strategiesaiming atimproving cardiomyocyte survivalrate and blood circulation highly encouraged, which is ableto reduce drug-induced cardiomyocyte necrosis and subsequent ischemic-related permanent damage.^9^

 Among toxic metabolites of cyclophosphamide, acrolein is accused of being the main toxic agent, which is responsible for cardiomyocyte damage. Several chemical and herbal compounds have been investigated and introduced to reduce or prevent cardiac complications caused by cyclophosphamide mainly by inhibiting oxidative/nitrosative stress and inflammatory responses. However, the anti-angiogenic effects of toxic metabolites of cyclophosphamide, such as acrolein, have largely remained to be determined. Given the negative effects caused by the anti-angiogenic properties of acrolein, in this study, the targeted therapy against post-transplant cardiomyopathy has been also emphasized. In this regard, the application of cardiomyocyte survival factors for regenerative strategies and endogenous cardiomyocyte proliferation, as well as angiogenic stimuli, e.g., angiogenic factors, in the face of acrolein’s anti-angiogenic effect would be more appreciated.^9^

## Conclusion and Future Prospective

 To date, the management of cyclophosphamide-induced cardiotoxicity appears as a significant challenge in clinical settings. Although the acrolein-related anti-angiogenic feature is considered a positive aspect in favor of chemotherapy, targeting acrolein’s anti-angiogenic effect could be regarded as a great promising approach to preserve cardiac function and improve clinical outcomes in the field of transplant medicine. In this respect, upcoming research is required to explore various angiogenesis stimulants using novel drug delivery systems, e.g., exosome-base therapy to inhibit acrolein-related negative effects without influencing other physiological processes. Additionally, long-term pre-clinical and clinical studies are requested to assess the safety and efficacy of these pharmacotherapies in the post-HSCT setting. Together, the prospect of stimulating angiogenesis and concurrent cardiac repair through targeted therapies opens up new avenues for improving the quality of life for transplant recipients.

 Despite the clinical importance of toxicity induced by alkylating agents, in comparison with anthracycline drug class, e.g., doxorubicin, there is limited data to address novel therapeutic options for better management of this health issue. Regarding the molecular mechanisms, there is also limited data about nrf2/ARE-related signaling pathways to discover further therapeutic targets against induced cytotoxicity.

## Acknowledgements

 The authors would like to thank the cooperation of the Clinical Research Development Unit, Shahid Madani Hospital, Tabriz, Iran in conducting of this research.

## Competing Interests

 The authors declared that they have no conflict of interest regarding this work.

## Ethical Approval

 Not applicable.
